# LKB1 Regulates Vascular Macrophage Functions in Atherosclerosis

**DOI:** 10.3389/fphar.2021.810224

**Published:** 2021-12-15

**Authors:** Xuewen Wang, Ziwei Liang, Hong Xiang, Yanqiu Li, Shuhua Chen, Hongwei Lu

**Affiliations:** ^1^ Health Management Center, The Third Xiangya Hospital of Central South University, Changsha, China; ^2^ Department of Cardiology, The Third Xiangya Hospital of Central South University, Changsha, China; ^3^ Department of Clinical Laboratory, Yueyang people’s Hospital, Yueyang, China; ^4^ Center for Experimental Medicine, The Third Xiangya Hospital of Central South University, Changsha, China; ^5^ Department of Biochemistry, School of Life Sciences of Central South University, Changsha, China

**Keywords:** liver kinase B1, atherosclerosis, macrophage function, AMPK, inflammatory

## Abstract

Liver kinase B1 (LKB1) is known to shape the regulation of macrophage function by participating in multiple processes including cell metabolism, growth, and polarization. However, whether LKB1 also affects the functional plasticity of macrophages in atherosclerosis has not attracted much attention. Abnormal macrophage function is a pathophysiological hallmark of atherosclerosis, characterized by the formation of foam cells and the maintenance of vascular inflammation. Mounting evidence supports that LKB1 plays a vital role in the regulation of macrophage function in atherosclerosis, including affecting lipid metabolism reprogramming, inflammation, endoplasmic reticulum stress, and autophagy in macrophages. Thus, decreased expression of LKB1 in atherosclerosis aggravates vascular injury by inducing excessive lipid deposition in macrophages and the formation of foam cells. To systematically understand the role and potential mechanism of LKB1 in regulating macrophage functions in atherosclerosis, this review summarizes the relevant data in this regard, hoping to provide new ideas for the prevention and treatment of atherosclerosis.

## Introduction

Atherosclerosis is a systemic vascular disease, which is the pathological basis of cardiovascular and cerebrovascular diseases such as coronary heart disease and stroke (Ibanez et al., 2021; Shea et al., 2021). With the gradual increase in prevalence, atherosclerosis-related diseases have brought a heavy burden to patients and society, and become a major risk of death (Valanti et al., 2021). Atherosclerotic lesions are characterized by lipid deposition in the arterial intima, focal fibrosis, inflammation, and intimal plaque formation (Nakagawa et al., 2021). In the early stage of atherosclerosis, circulating chemokines derived from blood monocytes increase, which promote the migration of cells to the intima to become macrophages (Cochain and Zernecke, 2017). Meanwhile, a substantial amount of low-density lipoprotein (LDL) is deposited in the arterial wall and is oxidized into oxidized LDL (Ox- LDL). This oxidized form of LDL cannot be recognized by the LDL receptors and internalized into cells for decomposition but is mainly recognized and taken up by macrophages through the scavenger receptors (SR) (Shen et al., 2020). Cholesterol-laden macrophages then turn into foam cells, whose formation is a key marker of atherosclerotic plaque development (Bäck et al., 2019; Xia et al., 2020).

In addition to the formation and accumulation of foam cells, sustained inflammatory response is another indispensable contributor to atherosclerotic lesion (Jeon et al., 2020). As the main innate immune cells, macrophages play a key role in maintaining the inflammatory response of blood vessels and regulating the stability of atherosclerotic plaques by producing pro-inflammatory cytokines (Kim et al., 2021). Moreover, macrophages promote the development of atherosclerosis in other ways, such as through endoplasmic reticulum (ER) stress and autophagy (Zhou et al., 2021). Although the exact mechanism behind macrophage regulation of atherosclerosis is unclear, certain kinases seem to hold the key to determine macrophage phenotype and function.

Liver kinase B1 (LKB1) is a serine/threonine kinase that is widely present in various tissues and cells and regulates cell proliferation, metabolism, polarity, and migration (Zhang Y. et al., 2021). LKB1 mainly acts by phosphorylating and activating adenosine monophosphate-activated protein kinase (AMPK) (Hollstein et al., 2019). Through AMPK, LKB1 regulates lipid metabolism, glycolysis, and other metabolic pathways to maintain the phenotype and function of normal cells (Zhang Y. et al., 2021; Molina et al., 2021). LKB1 is also a negative regulator of inflammatory response (Wu et al., 2021). Studies have shown that LKB1 exerts anti-inflammatory effects by activating AMPK in macrophages to inhibit the production of pro-inflammatory mediators and chemokines (Filippov et al., 2013). Moreover, LKB1 may participate in the regulation of ER stress and autophagy of macrophages through other pathways (Khayati et al., 2020; Zuo et al., 2020). With the deepening of understanding, the pathophysiological value of LKB1 in cardiovascular and metabolic diseases has attracted much attention (Liang et al., 2021; Liu et al., 2021). It has been observed that LKB1 expression is down-regulated in atherosclerotic macrophages and is involved in the regulation of atherosclerosis (Liu et al., 2017). Given that the particular regulatory importance of LKB1 in macrophage function in atherosclerosis has not been systematically described, here we review the role and possible mechanism of LKB1 in shaping macrophage function to reveal the potential target for the treatment of atherosclerosis.

### Functional Plasticity of Macrophages in Atherosclerosis

The etiology of atherosclerosis is more complex than previously thought, and its risk factors include hypertension, hyperlipidemia, heavy smoking, diabetes, and genetic predisposition (Lien et al., 2020; Wang and Ge, 2021; Zhao et al., 2021). Generally, different factors interact and converge to affect the formation of foam cells and the maintenance of vascular inflammation, thereby promoting the occurrence of atherosclerosis (Poznyak et al., 2020a). There is no doubt that the phenotypic and functional changes of macrophage are the hub of the pathological process of atherosclerosis (Zhang et al., 2019). The phenotypic and functional plasticity of atherosclerotic macrophages mainly involves lipid metabolism reprogramming, maintenance of inflammatory response, ER stress, and autophagy (Sukhorukov et al., 2020a; Cui et al., 2021). Among these, how lipid metabolism affects the phenotype and function of atherosclerotic macrophage has been extensively studied (Zhang Y. X. et al., 2021; Kotlyarov and Kotlyarova, 2021). Under normal conditions, macrophages can process intracellular lipids through uptake, synthesis, storage, and outflow to ensure the dynamic equilibrium of lipid metabolism (Sukhorukov et al., 2020b). However, when lipid intake exceeds the tolerance limit of macrophages, or relevant receptors and pathways are dysfunctional, the lipid metabolism of the cells will be disrupted and reprogrammed, resulting in the transformation of macrophages into foam cells and then the occurrence of atherosclerosis (Poznyak et al., 2020a).

The vascular inflammation of atherosclerosis is also one of the current research hotspots. Macrophages participate in atherosclerotic lesions by releasing pro-inflammatory cytokines and anti-inflammatory cytokines to maintain vascular inflammation (Cai D. et al., 2021). Excessive infiltration of macrophages will not only lead to focal arterial inflammation, but also increase the risk of systemic vascular inflammation; either way it will accelerate the progression of atherosclerosis (Lavin et al., 2020). Macrophage polarization is involved in maintaining vascular inflammation and has been shown to be necessary for the occurrence and development of atherosclerosis (Liu et al., 2020). Under different conditions, macrophages polarize into different subtypes to participate in the inflammatory response. Macrophages in atherosclerosis may polarize into the classically activated macrophage (M1) and alternatively activated macrophage (M2) phenotypes (Murray et al., 2014). The M2 phenotype can be further divided into subtypes M2a, M2b, M2c, and M2d (Colin et al., 2014). In addition, macrophages in atherosclerosis can differentiate into Mox, Mhem, M4, and M(Hb) phenotypes under the stimulation of certain factors. According to their functionality, M1, Mox, and M4 mainly have pro-inflammatory effects and promote the formation of atherosclerosis, while M2, Mhem, and M(Hb) can produce anti-inflammatory effects and prevent the formation of foam cells (Colin et al., 2014; Jinnouchi et al., 2020). M1 and M2 macrophages are the most important polarization subtypes in atherosclerosis. In the initial stage of vascular inflammation, circulating monocytes are recruited into vascular tissues, and are mainly polarized into M1 under the stimulation of pro-inflammatory factors. M1 macrophages further secrete tumor necrosis factor-α (TNF-α), (human) β-Interleukin 1 (IL-1β), interleukin-6 (IL-6), Interferon-γ (IFN-γ), and other pro-inflammatory mediators, which in turn aggravate vascular inflammation (Saha et al., 2017). Relatively, macrophages polarize into M2 during the self-repair of blood vessels, and inhibit M1-mediated vascular inflammation by up-regulating transforming growth factor-β (TGF- β), interleukin-10 (IL-10), and other anti-inflammatory cytokines (Murray, 2016). Although M1 and M2 can be seen in both early and late atherosclerotic lesions, as atherosclerosis progresses, the number of M1 gradually increases and the number of M2 gradually decreases (Bisgaard et al., 2016).

ER stress in macrophages is also closely related to the occurrence of atherosclerosis (Yang B. et al., 2020). ER stress aggravates atherosclerosis by inducing foam cell formation, apoptosis, and the release of pro-inflammatory cytokines (Tian et al., 2019; Wang T. et al., 2020). Increased expression of the ER stress-associated apoptosis signaling molecule C/EBP homoiogous protein (CHOP) indicates macrophage apoptosis (Yang et al., 2020b). Pathologically, macrophage apoptosis instigates the formation of inflammatory necrotic core, which is not conducive to the stability of advanced atherosclerotic plaques and thus underlies plaque rupture (Qiu et al., 2021).

On the contrary, autophagy of macrophages can delay cell death by repairing damaged macrophages, thereby protecting against atherosclerosis. Also, the autophagic machinery helps nearby phagocytes to effectively eliminate damaged cells (Liao et al., 2012). Thus, it is expected that dysregulated macrophage autophagy will contribute to the occurrence and development of atherosclerosis through promoting dyslipidemia, foam cell formation, and inflammation (Kumar et al., 2021; Shan et al., 2021). However, it has been observed that macrophage autophagy has a dual regulatory effect on the inflammatory response (Zhang J. et al., 2021). Under normal circumstances, autophagy can effectively inhibit the excessive activation of inflammasomes, thereby alleviating severe inflammatory response. However, when this self-digesting mechanism is inhibited or damaged, cathepsin will leak from the damaged lysosomes, further activates the inflammasomes, thus exacerbating the inflammatory response (Takahama et al., 2018). Generally, functional changes of lipid metabolism reprogramming, inflammation maintenance, ER stress and autophagy in macrophages are dynamic and interconnected, but any abnormal alterations in these cellular functions may eventually lead to atherosclerotic progression at multiple levels.

### LKB1 and Lipid Metabolism Reprogramming of Macrophages in Atherosclerosis

Lipid metabolism reprogramming is one of the main causes of early atherosclerosis formation. There are many types of lipids in the body, among which total cholesterol (TC) and triglyceride (TG) aremainly related to atherosclerosis. TC consists of free cholesterol (FC) and cholesterol ester (CE) (Cai Y. et al., 2021). Due to poor water solubility, lipids often exist in the form of highly soluble lipoproteins. The main lipoproteins closely related to atherosclerosis are LDL and chemically modified LDL, such as Ox-LDL (Malekmohammad et al., 2021). The circulating LDL binds to the LDL receptors on normal cell membranes, but ox-LDL does not. Ox- LDL is recognized by SR on the surface membrane of macrophages and taken up into the cell for metabolism. This internalization and process involves SR class A type 1 (SR-A1), SR class B type 1(SR-B1), ATP-binding cassette transporter A1 (ABCA1), and adenosine triphosphate-binding cassette (ABC) transporter G1 (ABCG1) (Jia et al., 2019; Kobayashi et al., 2021; Tao et al., 2021). Lipids in macrophages are normally metabolized through uptake, synthesis, storage, and outflow (Chistiakov et al., 2017). This metabolic mechanism is essential for maintaining the steady state of lipid metabolism. The imbalance of lipid metabolism will initiates the reprogramming of lipid metabolism and the differentiation of macrophages into foam cells, which is a prerequisite for the formation of atherosclerosis (Poznyak et al., 2020a; Poznyak et al., 2020b).

**FIGURE 1:**
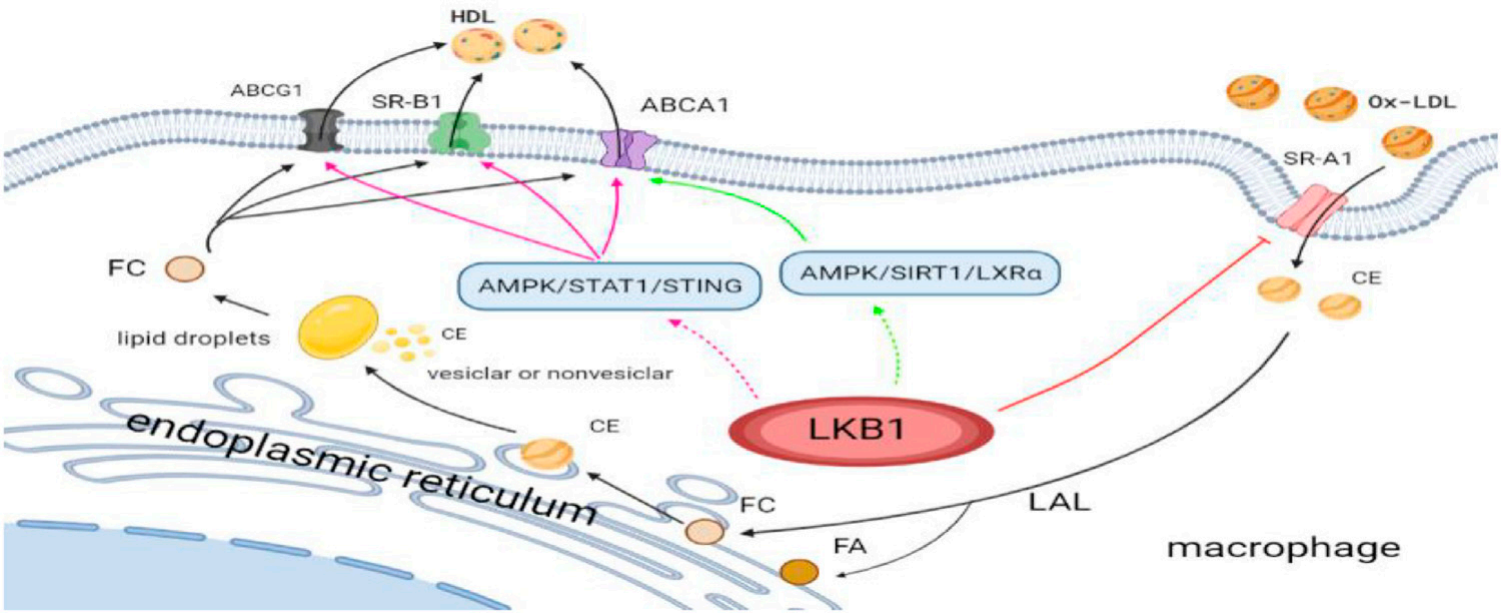
LKB1 and lipid metabolism reprogramming of macrophages in atherosclerosis. Ox-LDL, oxidized low-density lipoprotein; SR-A1, scavenger receptor class A type 1; CE, cholesterol ester; FA, fatty acid; FC, free cholesterol; LAL, lysosomal acid lipase; HDL, high-density lipoprotein; SR-B1, scavenger receptor class B type 1; ABCG1, adenosine triphophate (ATP)-binding cassette (ABC) transporter G1; ABCA1, ATP-binding cassette transporter A1.

LKB1 can inhibit the formation of atherosclerosis by reprogramming lipid metabolism of macrophages. On the one hand, LKB1 can affect the lipid uptake of macrophages. Macrophage membrane molecule SR-A is closely related to pathological intracellular lipid deposition, and its primary function is to recognize and uptake Ox-LDL and mediate the formation of foam cells. However, LKB1, as the upstream kinase of SR-A, can phosphorylate SR-A for degradation (Liu et al., 2017). It has been found that in the process of atherosclerotic plaque progression, the membrane expression of SR-A is high, while the expression of LKB1 in macrophages is decreased (Lin et al., 2016; Liu et al., 2017). These findings indicate that in the absence of phosphorylation and degradation of SR-A, SR-A can increase pathological lipid deposition and promote foam cell formation. On the other hand, LKB1 may affect lipid efflux from macrophages. Macrophages lipid efflux is dependent on the expression of ABCA1, ABCG1, and SR-B1 on the membrane surface. The high expression of these transporters and receptors will facilitate lipid efflux and reduce foam cell formation. It has been confirmed that the activation of AMPK/SIRT1/LXRα and AMPK/STAT1/STING signaling pathways in macrophages can up-regulate the expressions of ABCA1, ABCG1, and SR-B1, promote intracellular cholesterol outflow, reduce lipid accumulation, and ultimately prevent the occurrence of atherosclerosis (Lin et al., 2015; Cai D. et al., 2021). Interestingly, LKB1 is the main upstream kinase of AMPK, so it may participate in cellular lipid metabolism by activating AMPK (Xi et al., 2018). Nevertheless, more evidence is needed to evaluate that macrophage LKB1 participates in intracellular lipid outflow and negatively regulates atherosclerotic plaque formation by activating AMPK/SIRT1/LXR α and AMPK/STAT1/STING signaling pathways (Figure1).

### LKB1 and Maintenance of Macrophage Inflammatory Response in Atherosclerosis

Inflammatory immune response is an essential part of the pathogenesis of atherosclerosis (Liao et al., 2021). More specifically, vascular inflammation is the main driving force for the formation and growth of atherosclerotic lesions and the development of unstable ruptured plaques (Romanenko et al., 2021). Mechanistically, inflammatory response mainly relies on various cytokines and mediators to participate in the whole process of atherosclerosis development. Especially in the late stage of atherosclerosis, pro-inflammatory cytokines cause abnormal functions of macrophages, endothelial cells, and lymphocytes, thus accelerating the destruction of plaques (Poznyak et al., 2021). Cytokines are synthesized by various immune cells and secreted in autocrine and paracrine manners. As the most abundant immune cells in atherosclerotic lesions, macrophages have a vital role in the entire disease process from the onset of the lesion to the rupture of the plaque (Chen et al., 2020). Under the long-term effect of various interactive factors such as dyslipidemia, the arterial intimal damage is aggravated, the expression of adhesion factors increases, the number of monocytes attached to the endothelial cells gradually increases, and the number of intimal macrophage increases (Shen et al., 2020). Atherogenic macrophages produce and secrete a variety of pro-inflammatory cytokines [i.e., TNF-α, IL-1β, interleukin-18 (IL-18), IL-6, interleukin-23 (IL-23)] to maintain vascular inflammation and promote the growth of atherosclerotic plaques (Poznyak et al., 2021).

LKB1 is closely related to the release of a variety of pro-inflammatory cytokines (Wang et al., 2019). It was found that activating LKB1/AMPK and LKB1/MARK2 signaling pathways can reduce the release of TNF- α, IL- 6, and IL- 1β from macrophages, thereby inhibiting the inflammatory response (Wu et al., 2018; Deng et al., 2020). AMPK is a cellular energy sensor, and its α subunit is mainly distributed in the heart, brain, and liver (Molina et al., 2021). Studies have shown that LKB1 down-regulates the expression of IL-1 β, IL-18, and IL-23 through AMPKα phosphorylation, thereby relieving the inflammatory damage at the affected sites (Liu et al., 2019; Guo et al., 2021). However, whether LKB1 inhibits the release of pro-inflammatory cytokines from macrophages through the Mitogen-activated protein kinase2 (MARK2) and AMPK signaling pathways and further regulates the process of atherosclerosis is not fully understood.

**FIGURE 2:**
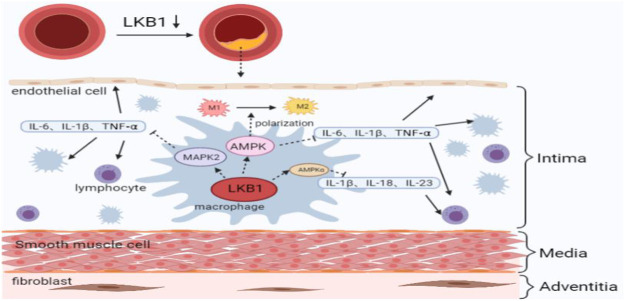
LKB1 and maintenance of macrophage inflammatory response in atherosclerosis. M1, classically activated macrophage; M2, alternatively activated macrophage; IL, interleukin; TNF, tumor necrosis factor; AMPK, adenosine 5′-monophosphate activated protein kinase; MAPK, mitogen-activated protein kinase.

In addition, as mentioned above, the ability of macrophages to orchestrate an inflammatory response is related to their polarization phenotypes. Different polarization phenotypes have distinct cytokine secretion profiles, which exhibit opposite effects on the formation of atherosclerosis. It has been reported that up-regulation of LKB1 expression can reduce macrophage infiltration and M1 polarization (Yang et al., 2020c). Furthermore, the LKB1/AMPK signaling pathway can promote the transformation of macrophages from M1 to M2, although further studies are needed to determine the involvement of LKB1 in the regulation of atherosclerotic inflammation by altering macrophage polarization phenotypes (Ji et al., 2018). While the metabolic signatures of M1 macrophages are increased glucose uptake and enhanced anaerobic glycolysis, activated M2 macrophages show significantly increased oxygen consumption through fatty acid oxidation and oxidative phosphorylation (Jha et al., 2015; Andrejeva and Rathmell, 2017). In view of this, it has been suggested that the metabolic transformations between glycolysis and mitochondrial oxidative phosphorylation are related to the direction of macrophage polarization (Mouton et al., 2020). Moreira et al. found that the LKB1 signaling pathway plays a vital role in the energy metabolism of macrophages, and the LKB1/AMPK signaling pathway regulates the transformation of energy metabolism from glycolysis to oxidative phosphorylation during inflammation (Moreira et al., 2015). This energy transformation regulated by LKB1 is likely to be the reason why macrophages polarize to M2 and participate in the atherosclerotic process. In any case, the energy transformation in the process of macrophage phenotype changes is worthy of further exploration (Figure 2).

### LKB1 and Macrophage ER Stress in Atherosclerosis

Various pathological conditions can lead to the accumulation of unfolded or misfolded proteins in the ER lumen. This phenomenon is called ER stress and usually causes ER dysfunction (Qiao et al., 2021). ER stress performs many pathophysiological functions through the activating transcription factor-6 (ATF-6), transmembrane inositol-requiring enzyme-1 (IRE1), protein kinase RNA-like ER kinase (PERK), and their downstream signaling pathways (Ren et al., 2021). Recent studies have found that ER stress plays a vital role in the occurrence and development of atherosclerosis, which is manifested as a significant upregulation of the ER stress marker CHOP in atherosclerotic plaque macrophages (Ma et al., 2020; Yang B. et al., 2020). It is precisely by mediating the ER stress of intravascular macrophages, endothelial cells, and smooth muscle cells that a variety of factors participate in and exacerbate the pathogenesis of atherosclerosis (Yang et al., 2020b). Among these intravascular cells, macrophage ER stress mainly aggravates atherosclerotic vascular damage by inducing foam cell formation and activating the apoptotic signaling pathway (Zahid et al., 2020).

Note that ER stress cross-regulates the production of pro-inflammatory cytokines, which are the main force for maintaining vascular inflammation (Chen et al., 2018). The synergistic effect of ER stress and inflammation substantially affects the formation and stability of dynamic atherosclerotic plaques, which is an essential condition for the development of atherosclerosis. In addition, excessive accumulation of FC in cells is one of the main causes of ER stress in macrophages (Zhu et al., 2008). ER stress in turn actively regulates lipid metabolism in macrophages by coordinating the uptake and outflow of lipids, but ultimately leads to an increase in lipid content in cells. This event clearly involves two mechanisms. On the one hand, ER stress up-regulates the expression of SR-A and the cluster of differentiation 36 (CD36) to increase lipid intake. On the other hand, ER stress increases the expression of CHOP and inhibits the expressions of ABCG1, ABCA1, and SR-B1, thereby reducing lipid efflux (Wang Z. et al., 2020). These two strategies work synergistically to effectively promote the transformation of macrophages into foam cells (Sukhorukov et al., 2020a).

LKB1 has the ability to negatively regulate the ER stress of macrophages, so it is an important part of the atherosclerotic process. It has been found that activation of LKB1/AMPK signaling can reduce ER stress and help improve cardiovascular diseases (Li et al., 2015; Zuo et al., 2020). As mentioned above, LKB1 affects the expression of SR such as SR-A1 on the surface of macrophages, regulates lipid metabolism, inhibits the formation of foam cells, and thus exerts strong anti-atherosclerotic effects. Therefore, the inhibition of ER stress by LKB1/AMPK signaling may be through regulation of lipid metabolism. However, it is not clear whether this effect is achieved through direct interaction with ER stress or caused by indirect and complex cross-regulation mechanisms. In addition, macrophages produce different pro-inflammatory cytokines to maintain the inflammatory response when ER stress occurs. For example, activation of the PERK/ATF4/CHOP signaling pathway promotes the release of TNF-α and IL-1β, and activation of IRE1α/JNK signaling pathway stimulates the release of IL-1β, IL-6, and IL-8 (Li D. et al., 2021; Song et al., 2021). However, activating the LKB1/AMPK signaling pathway robustly inhibits ER stress, reduces the production of pro-inflammatory cytokines, and alleviates cellular inflammatory responses (Rao et al., 2019). Therefore, LKB1 may block the release of pro-inflammatory cytokines by inhibiting the ER stress of macrophages, thus exhibiting anti-atherosclerotic effects.

In addition, there are some adaptive mechanisms related to ER stress, including ER-associated degradation and unfolded protein response (UPR). Moderate activation of these mechanisms can counteract excessive ER stress (Ren et al., 2021). UPR is an important regulatory response of the ER stress process, which refers to the degradation of unfolded or faulty proteins in the ER. Interestingly, LKB1/AMPK signaling pathway relieves ER stress by regulating UPR (Meares et al., 2011; Karunakaran et al., 2021). However, UPR may initiate apoptosis under prolonged ER stress. This is because excessive saturated fatty acids and FC in the ER activate CHOP through IRE1α/JNK, IRE1α/XBP1, ATF6/XBP1, and PERK/eIF2α/ATF4 pathways, thus inducing macrophage apoptosis (Tian et al., 2019). Macrophage apoptosis is the basis for the formation of atherosclerosis vulnerable plaques. There are reasons to believe that LKB1 can reduce macrophage apoptosis and slow the progression of atherosclerosis by inhibiting ER stress, but this needs to be verified.

**FIGURE 3:**
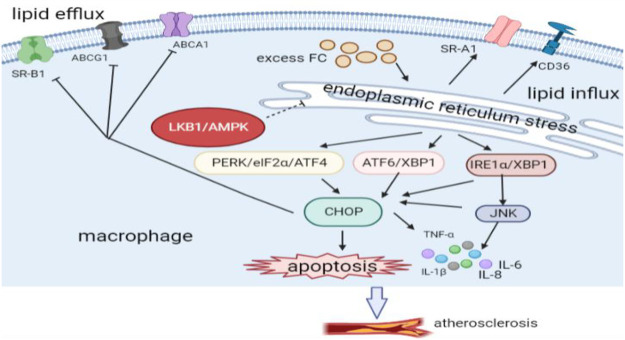
LKB1 and endoplasmic reticulum stress of macrophages in atherosclerosis. CD, Cluster differentiation; PERK, protein kinase RNA-like ER kinase; ATF4, activating transcription factor 4; ATF6, activating transcription factor 6; XBP1, the transcription factor X-box-binding protein 1; IRE1α, inositol-requiring protein 1α; JNK, c-JUN N-terminal kinase; CHOP, C/EBP homoiogous protein.

In general, the function of LKB1 is partially related to the ER stress of vascular macrophages. LKB1 can regulate the lipid metabolism of macrophages through the ER stress regulation of SR-A, ABCG1, ABCA1, and SR-B1. Furthermore, LKB1 regulates vascular inflammation and macrophage apoptosis by affecting ER stress. These regulatory mechanisms may work together and form an overlapping regulatory network in atherosclerosis, which needs to be further explored in the future (Figure 3).

### LKB1 and Macrophage Autophagy in Atherosclerosis

Macrophage autophagy seems to have a dual role in atherosclerosis: dysregulated autophagy promotes atherosclerosis, and moderate autophagy inhibits plaque progression (Zahid et al., 2021). Autophagy is a conservative degradation mode of cells, which is essential for maintaining the main functions of cell metabolism (Chen et al., 2021). When cells suffer from nutrient deficiency, oxidative stress, and organelle dysfunction, autophagy degrades unwanted materials through a lysosomal-dependent pathway to maintain cell homeostasis (Duan et al., 2020). Autophagy of macrophages play an anti-atherosclerotic effect by inhibiting the formation of foam cells and eliminating inflammation (Wang Z. et al., 2020; Tao et al., 2021). Also, autophagy promotes the transformation of foam cells into alternately activated macrophages to alleviate late-stage atherosclerosis (Robichaud et al., 2021). When autophagy is dysregulated, inflammasomes will be overactivated and promote the development of atherosclerosis (Razani et al., 2012). Even in advanced atherosclerosis, dysregulation of macrophage autophagy can increase the number of plaque lesions and the area of necrotic foci (He et al., 2020).

**FIGURE 4:**
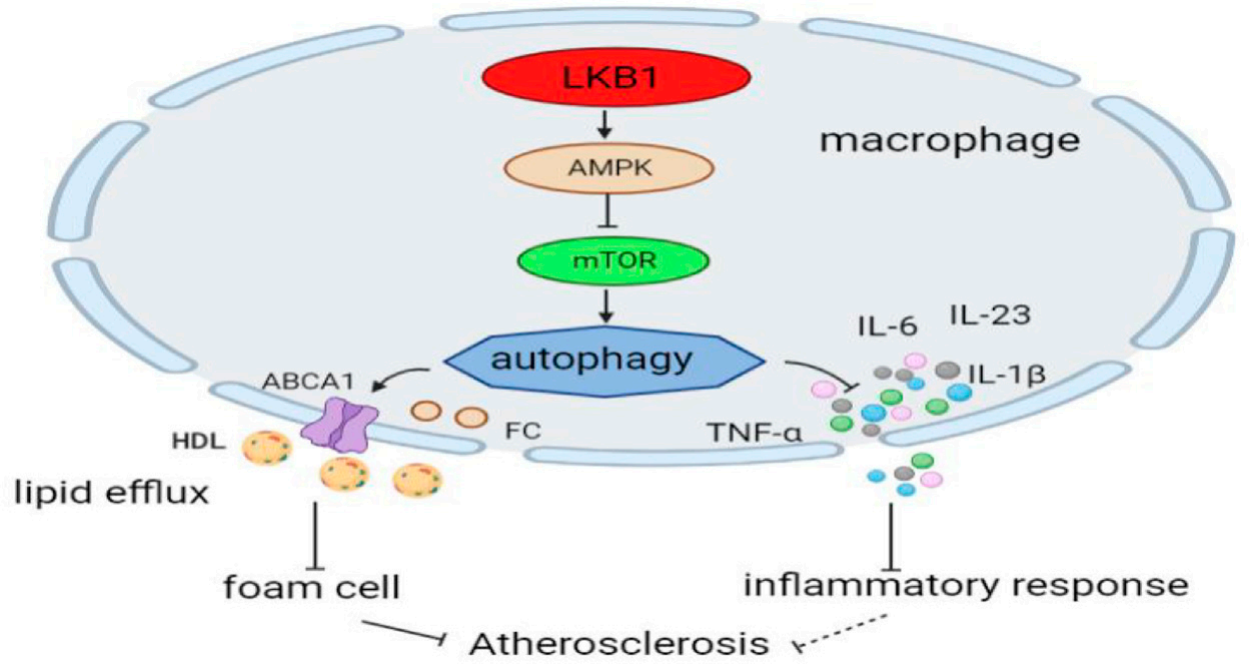
LKB1 and macrophage autophagy in atherosclerosis. Activation of the LKB1/AMPK/mTOR signaling pathway can induce autophagic activity in macrophages.

LKB1 affects the occurrence and development of atherosclerosis by regulating the autophagy of macrophages. On the one hand, LKB1 regulates lipid metabolism in macrophages by altering autophagy (Liang et al., 2021). The LKB1/AMPK/mTOR pathway can induce increased autophagy, inhibit lipid synthesis, and promote fatty acid oxidation (Li W. et al., 2021). Moreover, this pathway in macrophages can improve intracellular cholesterol accumulation by promoting cholesterol outflow and reduce the formation of foam cells in atherosclerosis (Li et al., 2019). On the other hand, LKB1 suppresses inflammation by inducing autophagy in macrophages (Liu et al., 2019). The LKB1/AMPK/mTOR signaling pathway reduces the synthesis and release of pro-inflammatory cytokines, such as IL-6, IL-23, IL-1β, and TNF-α (Alexander et al., 2010; Li et al., 2019). However, whether LKB1 has a similar regulatory effect in atherosclerotic macrophages needs to be tested (Figure 4).

## Conclusion

**FIGURE 5:**
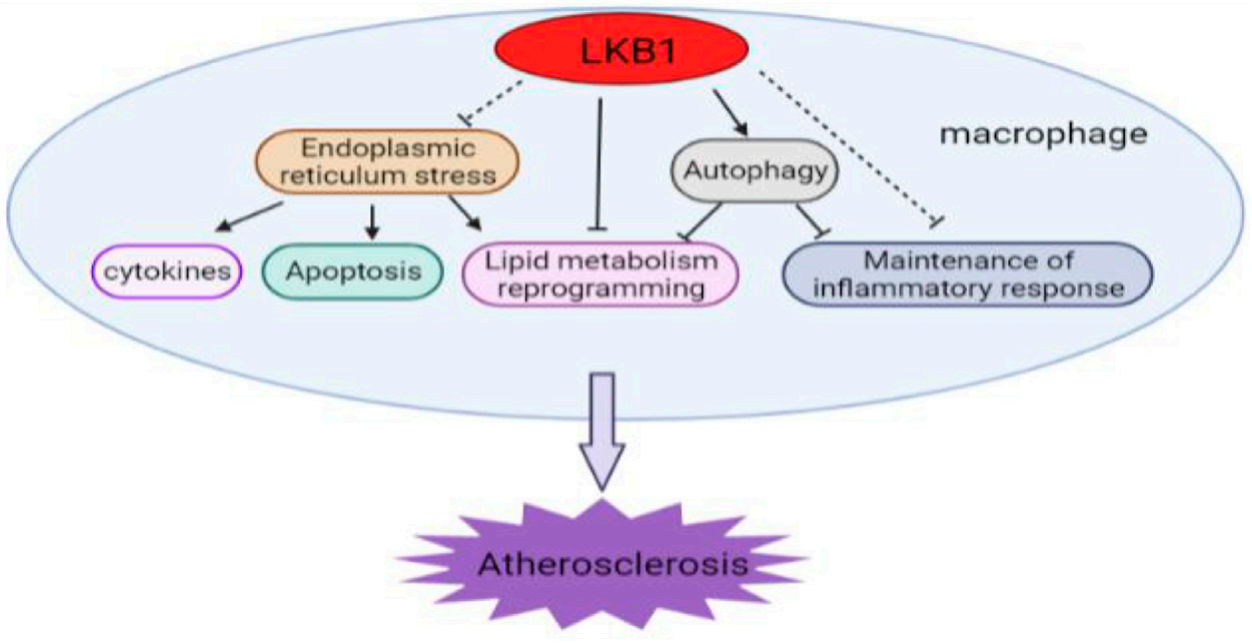
The diverse and complex roles of LKB1 in the development of atherosclerosis. LKB1 participates in multiple regulatory activities in vascular macrophages.

LKB1 is a serine-threonine kinase expressed in a variety of cells and is involved in regulating cell metabolism, polarity, and growth. LKB1 is a negative feedback regulator of atherosclerosis, which inhibits foam cell formation and vascular inflammation in many ways. LKB1’s regulation of macrophage lipid metabolism and its inhibition of foam cell formation are achieved by directly affecting the expression of SR or indirectly regulating autophagy. It can also regulate the release of pro-inflammatory cytokines in a variety of ways to inhibit vascular inflammation. Moreover, macrophage ER stress is closely related to the occurrence and development of atherosclerosis. LKB1 may participate in this disease process by affecting the ER stress of macrophages. Together, these different regulatory mechanisms of LKB1 may form an overlapping and complex network that determines the progression and outcome of atherosclerosis (Figure 5). Additionally, activation of LKB1 can alleviate atherosclerosis. This has been initially verified in clinical applications. For example, metformin, an important LKB1/AMPK activator, has been found to improve atherosclerosis (Vasamsetti et al., 2015; Ramachandran et al., 2018). Nevertheless, so far, our understanding of the exact regulatory mechanism of LKB1 in the functional changes of macrophage in atherosclerosis is rather limited. Given the important role of LKB1 in vascular macrophages, this regulator is worthy of further study and has great potential as a new target for the treatment of atherosclerosis.
